# Hydroclimate Variations in Central and Monsoonal Asia over the Past 700 Years

**DOI:** 10.1371/journal.pone.0102751

**Published:** 2014-08-13

**Authors:** Keyan Fang, Fahu Chen, Asok K. Sen, Nicole Davi, Wei Huang, Jinbao Li, Heikki Seppä

**Affiliations:** 1 Key Laboratory of Humid Subtropical Eco-geographical Process (Ministry of Education), College of Geographical Sciences, Fujian Normal University, Fuzhou, Fujian province, China; 2 Department of Geosciences and Geography, University of Helsinki, Helsinki city, Helsinki, Finland; 3 Key Laboratory of Western China's Environmental Systems (MOE), Lanzhou University, Lanzhou, Gansu province, China; 4 Richard G. Lugar Center for Renewable Energy and Department of Mathematical Sciences, Indiana University, Indianapolis, Indiana, United States of America; 5 Tree-Ring Lab, Lamont-Doherty Earth Observatory, Columbia University, Palisades, New York, United States of America; 6 Department of Geography, University of Hong Kong, Hong Kong city, Hong Kong, China; Chinese Academy of Sciences, China

## Abstract

Hydroclimate variations since 1300 in central and monsoonal Asia and their interplay on interannual and interdecadal timescales are investigated using the tree-ring based Palmer Drought Severity Index (PDSI) reconstructions. Both the interannual and interdecadal variations in both regions are closely to the Pacific Decadal Oscillation (PDO). On interannual timescale, the most robust correlations are observed between PDO and hydroclimate in central Asia. Interannual hydroclimate variations in central Asia are more significant during the warm periods with high solar irradiance, which is likely due to the enhanced variability of the eastern tropical Pacific Ocean, the high-frequency component of PDO, during the warm periods. We observe that the periods with significant interdecadal hydroclimate changes in central Asia often correspond to periods without significant interdecadal variability in monsoonal Asia, particularly before the 19^th^ century. The PDO-hydroclimate relationships appear to be bridged by the atmospheric circulation between central North Pacific Ocean and Tibetan Plateau, a key area of PDO. While, in some periods the atmospheric circulation between central North Pacific Ocean and monsoonal Asia may lead to significant interdecadal hydroclimate variations in monsoonal Asia.

## Introduction

Hydroclimate changes in central and monsoonal Asia have generated much concern due to their close linkages with water resources, critical for increasing population and economical development [1], [2]. Central and monsoonal Asia are strongly influenced by the westerlies and Asian summer monsoon. Hydroclimate changes in the two regions were out-of-phase or anti-phase on glacial-interglacial, millennial and centennial timescales [3], [4], [5]. Possible mechanisms related to this inverse relationship include the different responses of hydroclimate changes to external forcings (e.g. orbital changes and solar irradiance), the boundary conditions (e.g. ice volume), the internal ocean-atmospheric feedbacks (e.g. North Atlantic Oscillation (NAO) and Atlantic Multidecadal Oscillation (AMO)) and the regional topographic features (e.g. Tibetan Plateau), which can modulate the strength of the Asian summer monsoon and westerlies [3], [4], [5]. However, hydroclimate variations in central and monsoonal Asia at the interannual and interdecadal timescales and their relationships remain unclear [6], [7].

Tree-ring records are very useful for investigating the spatiotemporal climate changes extending before the industrial era on interannual and interdecadal timescales due to their annual resolution and wide spatial distribution, although the long-term variations on timescales longer than a century may not be well preserved due to the “segment length curse” problem in the developments of tree-ring chronology [8], [9]. The Palmer Drought Severity Index (PDSI) is a hydroclimate metric of the moisture deficiency relative to the normal condition and is scaled to have equal mean across space [10], [11]. Tree rings have been successfully used to reconstruct the PDSI to establish the PDSI atlas for the past 700 years over the entire monsoonal Asia and most of the central Asia, i.e. the Monsoon Asia Drought Atlas (MADA) [9]. In this study, we used the MADA to provide a comprehensive investigation of the hydroclimate variability of central and monsoonal Asia and their relationships.

## Data and Methods

### Climate data

**Figure 1:**
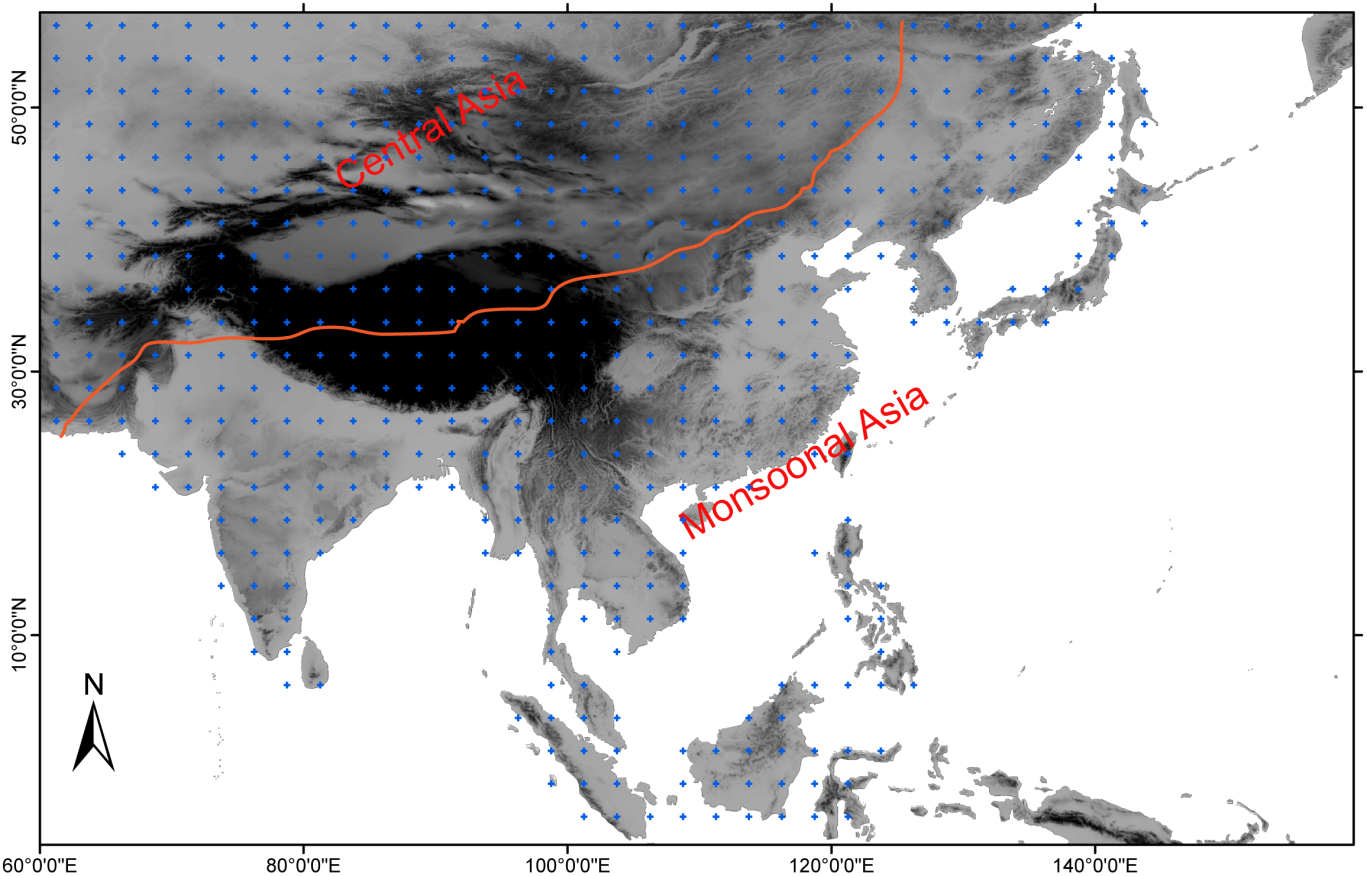
Location of the PDSI grids and the central and eastern Asia is delimited according to Chen et al. (2010).

The study region (60°E–160°E, 10°S – 60°N) covers regions of topographic complexity from the Tibetan Plateau to the low-lying regions such as the basins and river valleys (Figure 1). Precipitation generally decreases from the coast to the inland since the monsoon strength decays according to its distance to ocean [3]. The driest regions are the low-lying inland regions, e.g. the Taklimakan desert in Tarim Basin and the wettest region is the southern windward boundary of the Himalayan Mountains. Instrumental PDSI data were derived from a global dataset with 2.5°× 2.5° gridding from 1870 to 2005 [11]. The MADA consists of 534 grid cells of summer (June-July-August) PDSI records in central and monsoonal Asia, reconstructed from 327 tree-ring chronologies [9], most of which start since 1300 (504 grids). The point-by-point regression method was employed to locate the nearby tree-ring chronologies according to a search radius [9]. The PDSI data in regions with sparse tree-ring chronologies were reconstructed from remote regions and thus may contain some biases. For example, there are limitations in recovering the drought patterns in southern China, central Inner Mongolia and south central Mongolia where limited tree-ring chronologies are available [12]. The MADA is normally distributed with extremely dry (<−4) and wet (>4) PDSI values accounting for only a small percentages [12]. This indicates that the reconstructions are reasonable for spatiotemporal comparisons [13]. All the PDSI reconstructions were detrended by fitting a 150-year spline to remove the centennial or multi-centennial variations and highlight the interdecadal variations.

### Analytical methods

We investigate the percentage of areas affected by drought over the past 700 years, i.e. the Drought Area Index (DAI). The DAI is calculated as a ratio between the number of PDSI grids exceeding a threshold value and the sum of the PDSI grids of a given year. That is, high DAI indicates enlarged dry areas, and vice versa. This index has been applied in tree-ring based reconstructions in western North America [14]. The DAI pays special attention on the spatial features of the hydroclimate changes. As is known, the long-term climate changes in the tree-ring based reconstructions may be removed in the curve-fitting standardization process. While the spatial features of the DAI can show some long-term changes even if the low-frequency variations of the reconstructions for individual grids are removed. That is, the long-frequency information of the spatial coverage of the droughts can be better recovered relative to the amplitudes of the reconstructed droughts using the curve-fitting standardization method. In this study, we tested the use of the threshold values of lower than −1 and −2 that result in similar results and we thus only shown the DAI variations according to the threshold value lower than −1. We herein calculated the DAI for the arid central Asia and the monsoonal Asia [3], separately, due to the different climate regimes between them (Figure 1). The spectral properties of these time series were detected using the multi-taper method (MTM) that is particularly efficient for short time series [15]. We applied the wavelet analysis based on a Morlet function to transform the DAI series into a time-frequency profile [16], [17]. We additionally calculate the Cross Wavelet Transform (XWT) and Wavelet Coherence (WTC) of the Pacific decadal oscillation (PDO) and DAI in central and monsoonal Asia. The XWT identifies the common power, whereas WTC gives a measure of local correlation between two time series in the time-frequency plane. The relative phase between the two time series can also be discerned from the XWT or WTC. In order to further investigate the frequency-dependent relationships between climate patterns, a reciprocal pair of low-pass and high-pass Gaussian filters were also employed to separate low-frequency climate signals greater than 8 years from the high-frequency variations shorter than 8 years.

To explore the linkages between hydroclimate and sea surface temperature (SST), we applied the singular value decomposition (SVD) method [18] to detect the leading pattern between the reconstructed PDSI and the Extended Reconstruction Sea Surface Temperature (ERSST.v3b) [19] in the Indian and Pacific oceans (herein 20S-60N; 40E-90W) during the common period from 1854 to 2005. The SVD is an efficient method to identify coupled modes from the cross-covariance matrix between the two fields (herein reconstructed PDSI and ERSST) according to their importance (the portion of explained variances) [18]. In addition, we calculated the spatial correlations between MADA and the instrumental PDO indices (http://jisao.washington.edu/pdo/) [20] and the reconstructed PDO indices from tree rings [21].

### Hydroclimate variations in central and monsoonal Asia

#### DAI and its spectral properties

**Figure 2:**
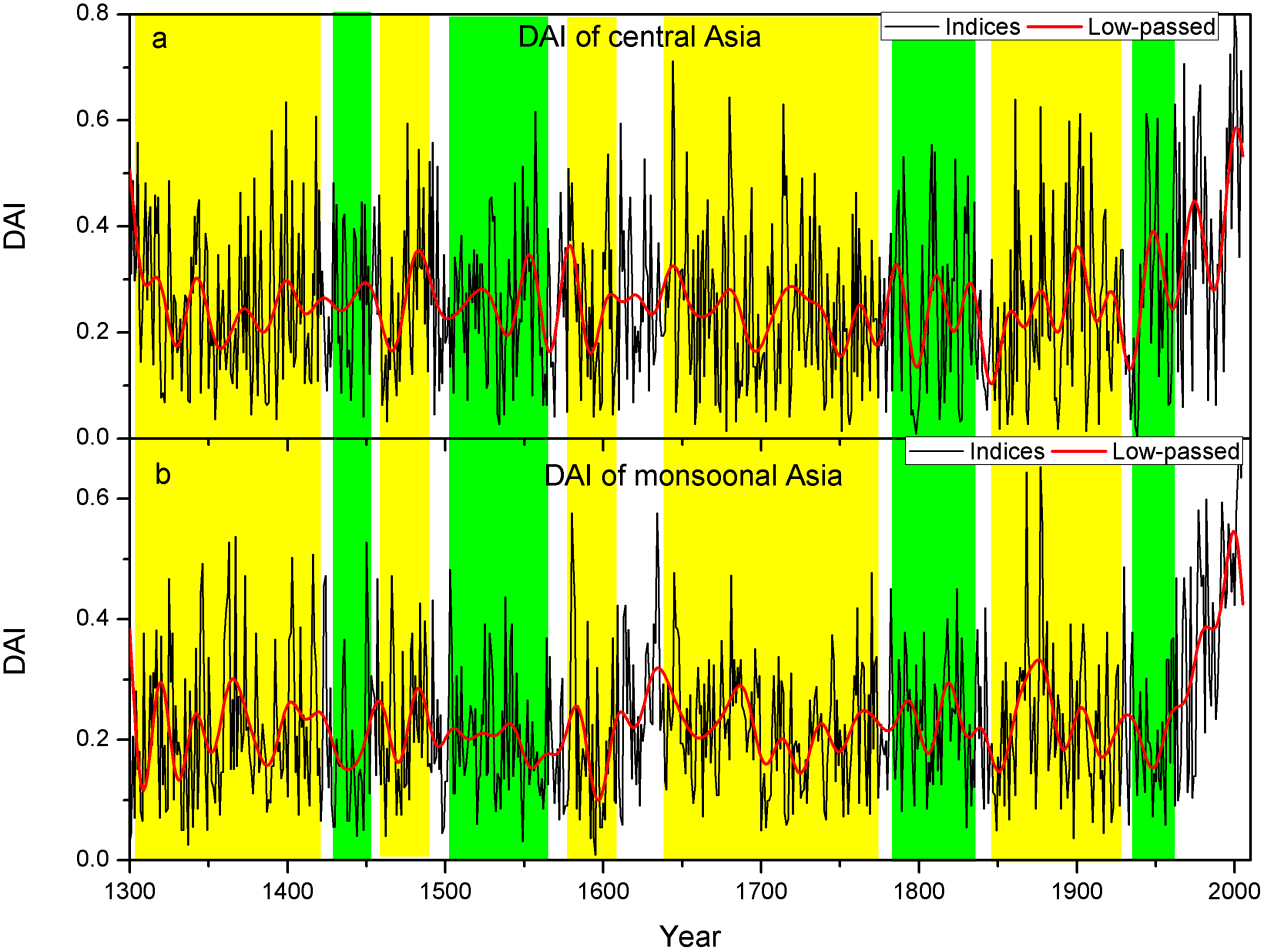
The drought area index (DAI) and the (red bold line) low-passed (lower than the cutoff frequency of 0.02) DAI for (a) central and (b) monsoonal Asia. The yellow shaded area indicates the in-phase decadal variations and the green shaded area indicates the anti-phase decadal variations between them.

**Figure 3:**
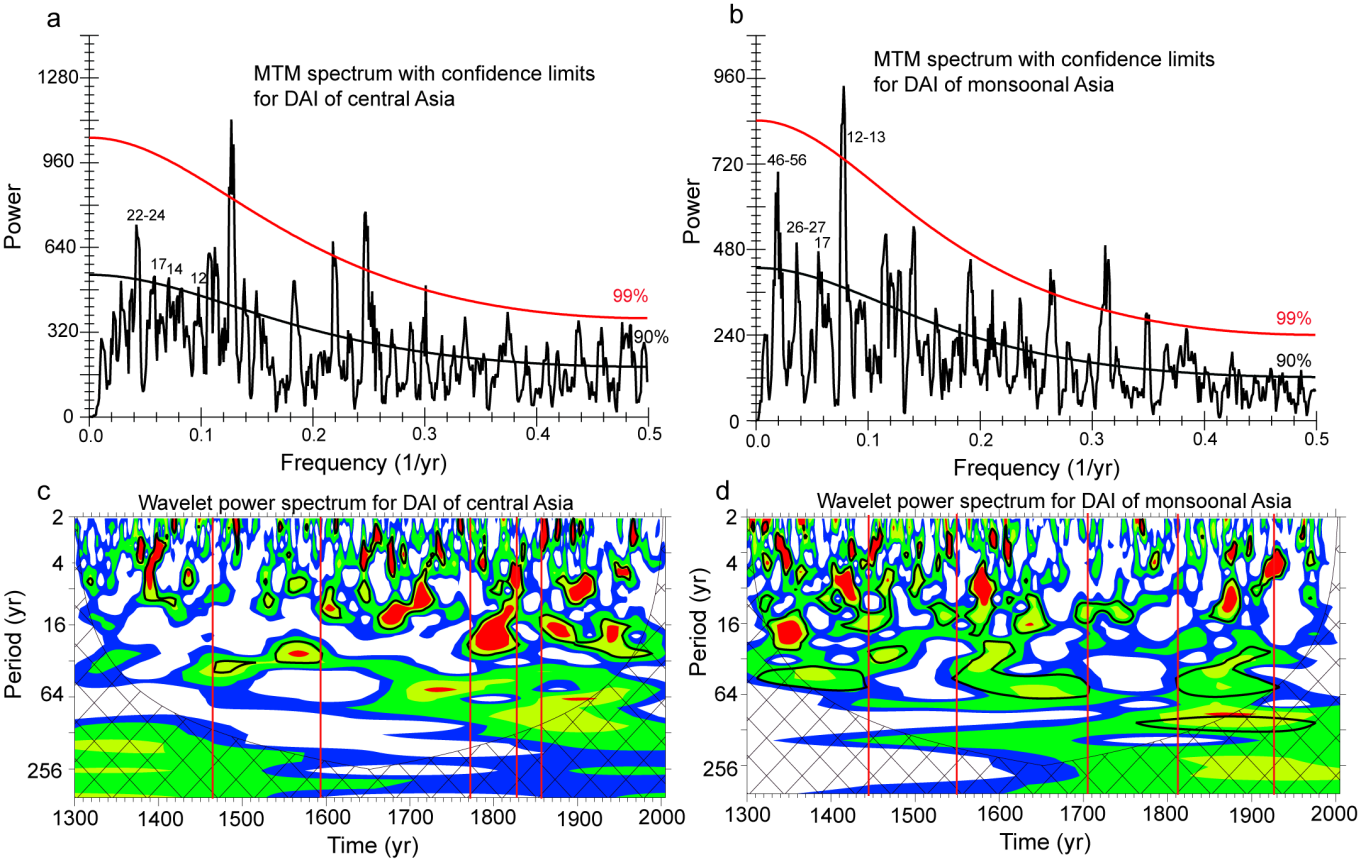
The multi-taper method (MTM) spectra of the drought area index (DAI) of the (a) central Asia and the (b) monsoonal Asia, as well as the wavelet spectra of the DAI of the (c) central Asia and the (d) monsoonal Asia.

The prominent feature of the DAI series is the increasing trend of the dry area since the 1950s (Figure 2). This trend coincides with the drying trend since the 1950s [6], [22] and may be exaggerated due to the potential overestimation of the evapotranspiration under the global warming trend [11], [23]. As revealed by the MTM analyses (Figure 3a, 3b), significant spectral peaks at around 2–3 years in both central and monsoonal Asia may be dominated by tropospheric biennial oscillation (TBO) [24], which has been widely documented in many tree-ring based hydroclimatic reconstructions [25], [26], [27], [28], [29], [30]. Other significant cycles of 3–7 years in both study regions might be related to El Niño-Southern Oscillation (ENSO). The interdecadal cyclic pattern of 22–24, 26–27 and 46–56 years can be linked with the PDO [31]. Potential relationships with ENSO and PDO will be further discussed below. The significant 22-year cycle might also be associated with Hale's solar cycle, which is also evidenced in a previous reconstruction in northeastern Tibetan Plateau [27].

#### Relationships between hydroclimate in central and monsoonal Asia

According to the wavelet analyses, shifts in frequency bands between the presence and absence of the interdecadal changes for central Asia were identified in the 1460s, 1590s, 1770s, 1820s, and 1850s (Figure 3c). Similarly, in monsoonal Asia, we detected shifts at interdecadal timescale in the 1440s, 1550s, 1700s, 1810s and 1920s (Figure 3d). Significant interdecadal variations during the 1460s–1590s and 1770s–1820s were found in central Asia, while the interannual variations dominated monsoonal Asia over the corresponding periods of the 1440s–1550s and the 1700s–1810s. On the other hand, significant interannual variations were found before the 1460s, 1590s–1770s, and 1820s–1850s for central Asia, which corresponds to significantly interdecadal variations over corresponding periods before the 1440s, 1550s–1700s and 1810s–1920s in monsoonal Asia (Figure 3). Some frequency shifts of the hydroclimate changes appear to be triggered by extreme climate conditions. For example, the frequency shift at 1450s–1460s corresponds with a dry period in much of the study region (Figure 2), particularly in northeastern Tibetan Plateau [27], [29], [30], [32]. The different dominant frequency properties were more conspicuous in the early periods, particularly before the 19^th^ century. Most of the long-term tree-ring chronologies are located in the central Asia and the marginal areas of monsoonal Asia [9], suggesting that different frequency properties might focus on central Asia and the marginal areas of monsoonal Asia. Increased number of tree-ring chronologies after 19th century (Figure S1) can be associated with the reconstruction of increased spatial features, such as for the monsoon-dominated Indian subcontinent with only two chronologies available in recent centuries. Further reconstructions of hydroclimate changes with more old chronologies of relatively even distribution may help clarify this. Although our results do not indicate anti-phase variations in the mean hydroclimate conditions between central and monsoonal Asia at the interannual or interdecadal timescale, there is evidence of the inverse shifts of the dominant frequency properties of hydroclimate changes occurring at intervals from some decades to a century. Different frequency properties between central and monsoonal Asia suggest different hydroclimate regimes between two regions.

Since the DAI displayed frequency-dependent variations, we further calculated the 51-year running correlations between the DAI in central and monsoonal Asia at interannual and interdecadal timescales (Figure S2). The relationships between DAI in central and monsoonal Asia is unstable through time. We further calculated the WTC between DAI in both regions (Figure S3). The DAI variations at interdecadal timescale correlate with each other better than at interannual timescale. This agrees with the visual comparisons between DAI in central and monsoonal Asia (Figure 2). Significant correlations between DAI in both regions at interdecadal timescale correspond to peaks in the running correlations of the low-passed data (Figures S1 and S2). Unstable relationships between DAI in central and monsoonal Asia may be because the coupled ocean-atmospheric patterns dominating these regions vary through time. We therefore explore the linkages between DAI variations in both regions with the coupled ocean-atmosphere pattern in the following section.

### Linkages to the coupled ocean-atmosphere patterns

#### PDO and hydroclimate changes

**Figure 4:**
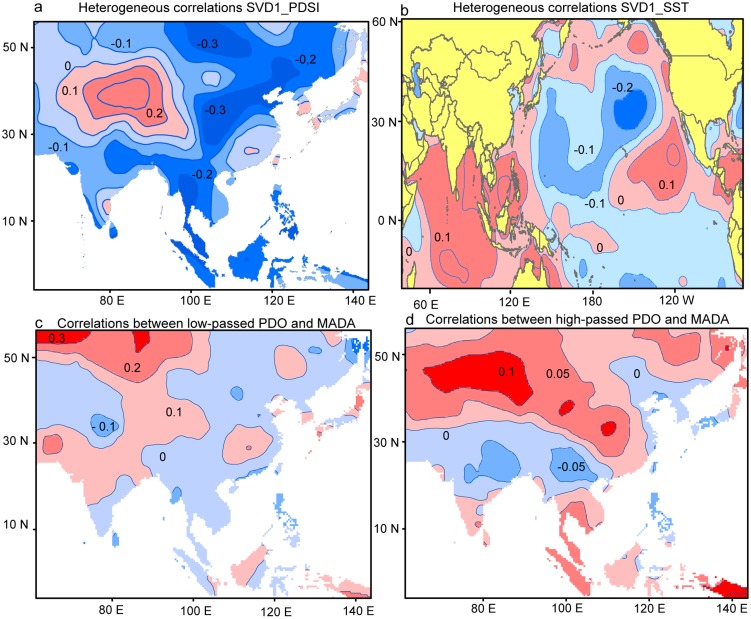
Heterogeneous correlations of the first leading SVD mode for the (a) reconstructed PDSI and (b) extended SST during the common period 1854–2005 and the (c) correlations between the low-passed PDO indices and the Monsoon Asia Drought Atlas (MADA) and the (d) correlations between the high-passed PDO and MADA over their common period period 1300–1996.

As for the first leading SVD mode between MADA and SST, significant (0.01 level) positive heterogeneous correlation with MADA are found in central Asia (around 30–40°N, 70–90°E), and are surrounded by horseshoe-like negative correlations in monsoonal Asia of eastern Mongolia, eastern China and Indochina (Figure 4a). Significant positive correlations with SST are seen in the eastern equatorial Pacific and oceans near western North America and negative correlations are located in the north Pacific (Figure 4b), corresponding to a positive phase of the PDO [20]. Positive correlations are also found in much of the Indian Ocean. This indicates that a positive (negative) PDO and warm (cold) Indian Ocean SST lead to pluvial (dry) conditions in central Asia and dryness (wetness) in monsoonal Asia. The close linkage to PDO with strong interdecadal variations may account for the above findings that the DAI in central and monsoonal Asia are higher correlated at the interdecadal timescale. Therefore our later discussions focus on the relationships with PDO. We further calculated the correlations between PDO and MADA at the interannual (Figure 4c) and interdecadal (Figure 4d) timescales. At both timescales, the spatial correlation pattern is similar to the heterogeneous map in Figure 4a, except that the negative correlations in monsoonal Asia are less significant. In addition, higher correlations are found over the tropical Asia at interannual timescale. The most significant, positive correlations since 1300 are found for central Asia, indicating that the relationships between PDO and hydroclimate variations in central Asia are most robust through time.

**Figure 5:**
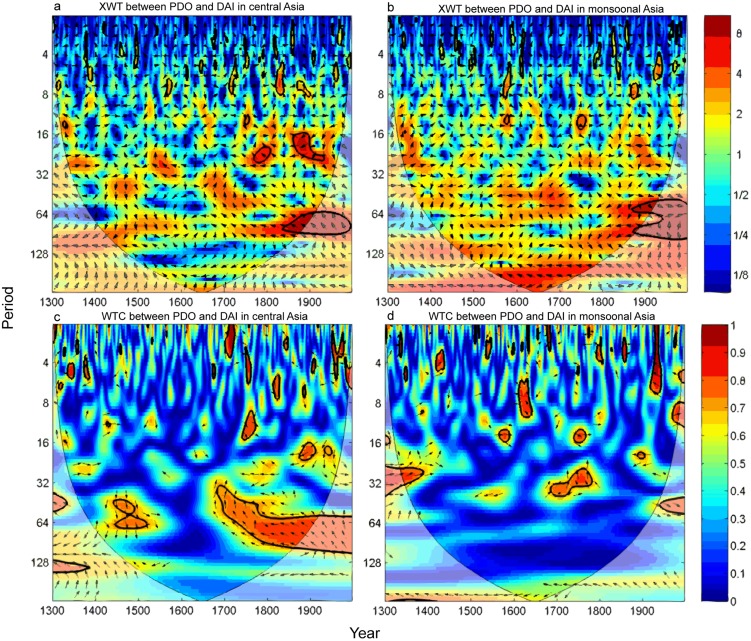
The cross wavelet transform (XWT) between PDO and DAI of (a) central and (b) monsoonal Asia, and the squared wavelet coherence (WTC) between PDO and DAI of (c) central and (d) monsoonal Asia. The significance level (*p*<0.05) is indicated by thick contours. The left arrows indicate anti-phase relationship, and right arrows indicate in-phase relationship between PDO and DAI.

To further detect the frequency-dependent relationships between PDO and hydroclimate through time in these two regions, we calculated the XWT and WTC between PDO and DAI in central and monsoonal Asia. There is significant common power between DAI in central Asia and PDO on interdecadal timescales, particularly from 1770 to 1820, and after 1850 (Figure 5a). These significant common powers correspond to significant interdecadal variations of DAI in the 1770s–1820s and since the 1850s. The WTC plot (Figure 5c) also indicates significant correlations between PDO and DAI in central Asia in the late 15^th^ century and since the 1850s on interdecadal timescale, which account for the significant interdecadal variations from the 1460s to 1590s and since 1850s in central Asia. There are significant correlations on interannual timescale before the 14^th^ century and from middle 17^th^ century to 18^th^ century, and since the 1850s, which correspond to the significantly interannual variations of the hydroclimate changes in central Asia. The good matches of the significant common powers and correlations between PDO and hydroclimate changes add in proofs to the close linkages between PDO and hydroclimate changes in central Asia. We also found correspondence between PDO and DAI in monsoonal Asia (Figure 5b and 5d). For example, significant correlation at the interdecadal timescale in the 14^th^ century may be related to the significant interdecadal variations prior to the 1450s. However, in comparison, the common power and local correlation between DAI in monsoonal Asia and PDO appears to be less significant.

**Figure 6:**
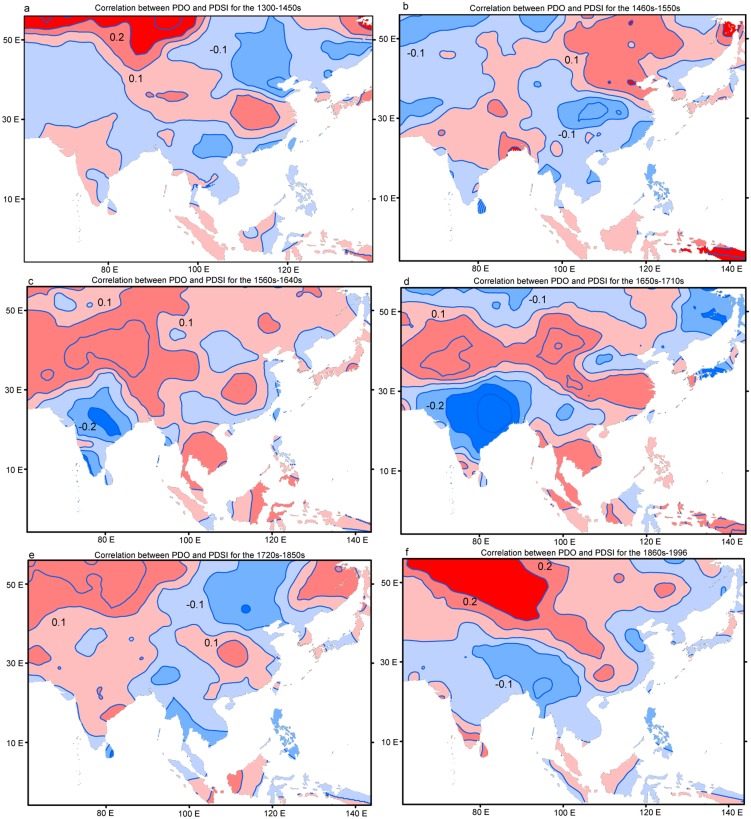
Correlations between the reconstructed PDO series and the reconstructed PDSI for the periods (a) 1300–1450s, (b) 1460s–1550s, (c) 1560s–1640s, (d) 1650s–1710s, (e) 1720s–1850s and (f) 1860s–1996.

We calculated the correlations between PDO and MADA over different subperiods generally corresponding to the different levels of solar irradiance [33]. Consistent with the above findings, we found that the correlations between PDO and MADA are more stable in central Asia. Positive correlations between PDO and PDSI in central Asia are significant in all periods except for the two periods with the minimum solar irradiance (Figure 6), i.e. the Spörer Minimum from 1460–1550 and the Maunder Minimum from 1645–1715 [33], and thus low temperature [34]. There is seemingly paradox as the interdecadal signal in central Asia is not conspicuous in warm periods. This is because the correlations during the subperiods are more likely to represent the relationships on interannual timescale as the interannual variations of these tree-ring based reconstructions in short subperiods generally account for larger variance. At interannual timescale, the DAI in central Asia is more conspicuous in warm periods. Taken together, significant interdecadal (interannual) hydroclimate changes in central Asia are modulated by the interdecadal (interannual) variations of PDO. The linkages between PDO and hydroclimate in central Asia is more conspicuous in warm periods high solar irradiance at the interannual timescale. While the relationships between PDO and DAI in monsoonal Asia is not stable.

#### Mechanisms for the interannual and interdecadal changes

The PDO is closely associated with ENSO and is considered the reddening signal (low-frequency) of ENSO with its interannual variability focusing on the tropical ocean and the interdecadal variability focusing on the north central Pacific Ocean [20], [35]. Therefore we treat the PDO-hydroclimate linkages at interannual timescale mainly via the tropical ocean similar as the ENSO-hydroclimate teleconnections, and the interdecadal PDO-hydroclimate linkages mainly via the North Pacific Ocean. On the interannual timescale, hydroclimate changes in central Asia can be influenced by propagating moisture from the Indian Ocean via the Iran Plateau [7], [36]. This northward moisture transportation can be enhanced by the anticyclone pattern in the Arabian Sea and the cyclone-like anomalies in the Iran Plateau and a trough in central Asia [36]. The anticyclone pattern over Arabian Sea can be strengthened when the SST of the tropical eastern Pacific Ocean is above average [37]. Since the variability of the tropical eastern Pacific Ocean tends to be strong (weak) in warm (cold) periods [38], the interannual variability of the hydroclimate changes in central Asia tends to be strengthened (weakened) in warm (cold) periods.

On the interdecadal timescale, the PDO-hydroclimate relationships may be linked via the atmospheric circulation between Tibetan Plateau and central North Pacific Ocean. An upward atmospheric circulation over Tibetan Plateau and a downward limb in the central North Pacific Ocean can lead to an increase in SST over the central North Pacific Ocean [39], [40], which corresponds to a negative phase of the PDO [20]. On the other hand, a rising motion over Tibetan Plateau can contribute to the drying tendency of central Asia by a subsiding flow of dry air [41], [42]. That is, during the positive (negative) phase of PDO, cold (warm) central North Pacific Ocean can be associated with downward (rising) atmospheric anomalies and thus more (less) air from Tibetan Plateau can be descended over central North Pacific Ocean, which leads to weak (enhanced) subsidences and thus wet (dry) climate over central Asia. This accounts for the significant interdecadal variations of hydroclimate changes in central Asia. On the other hand, PDO may be linked to monsoonal Asia instead of Tibetan Plateau, leading to significant interdecadal variations in monsoonal Asia. That is, a rising motion over monsoonal Asia when the monsoon is strong corresponds to a downward motion on central North Pacific Ocean and a high SST and thus a negative PDO. The negative correlations between PDO and hydroclimate in monsoonal Asia generally agree with the previous findings that suggested the negative correlation between PDO and the strength of Asian summer monsoon [7], [25], [35].

## Conclusions

We calculated DAI for central and monsoonal Asia and investigated their relationships and linkages with coupled ocean-atmosphere patterns. The presence (absence) of interdecadal variability in central Asia often correspond to periods without significant interdecadal variability in monsoonal Asia, particularly before the 19^th^ century, and vice versa, indicating different hydroclimate regimes between the two regions. This may be due to their linkages with PDO. The relationship between PDO and hydroclimate in central Asia is more robust than in monsoonal Asia. The positive correlations between the PDO and hydroclimate in central Asia are more robust in warm periods. This is because the interannual PDO variability, similar to the interannual ENSO variability, tends to be enhanced (weakened) in warm (cold) periods, resulting in high (low) interannual variability in central Asia during the warm (cold) periods prior to the industrial era. The significant interdecadal hydroclimate changes in central Asia are likely linked to the PDO via the atmospheric circulation between Tibetan Plateau and the central North Pacific Ocean. The significant interdecadal hydroclimate changes in monsoonal Asia may be linked to the atmospheric circulation between monsoonal Asia and the central North Pacific Ocean.

## Supporting Information

Figure S1 **The time-varying number of tree-ring chronologies used in MADA.**
(TIF)

Figure S2 **The 51-year running correlations between DAI in central and monsoonal Asia for the high-passed and low-passed data derived from a reciprocal pair of low-pass and high-pass Gaussian filters.**
(TIF)

Figure S3 **Squared wavelet coherence (WTC) of the drought area index (DAI) between central and monsoonal Asia.** The arrows pointing left (right) indicate anti-phase (in-phase) relationship. The areas with significance (*p*<0.05) relationships are indicated by thick contours.
(TIF)
