# Supplementary figures and images for: Hydroclimate Variations in Central and Monsoonal Asia over the Past 700 Years
Source: PLoS One. 2014 Aug 13;9(8):e102751. doi: 10.1371/journal.pone.0102751 (PMC4131873; doi:10.1371/journal.pone.0102751)

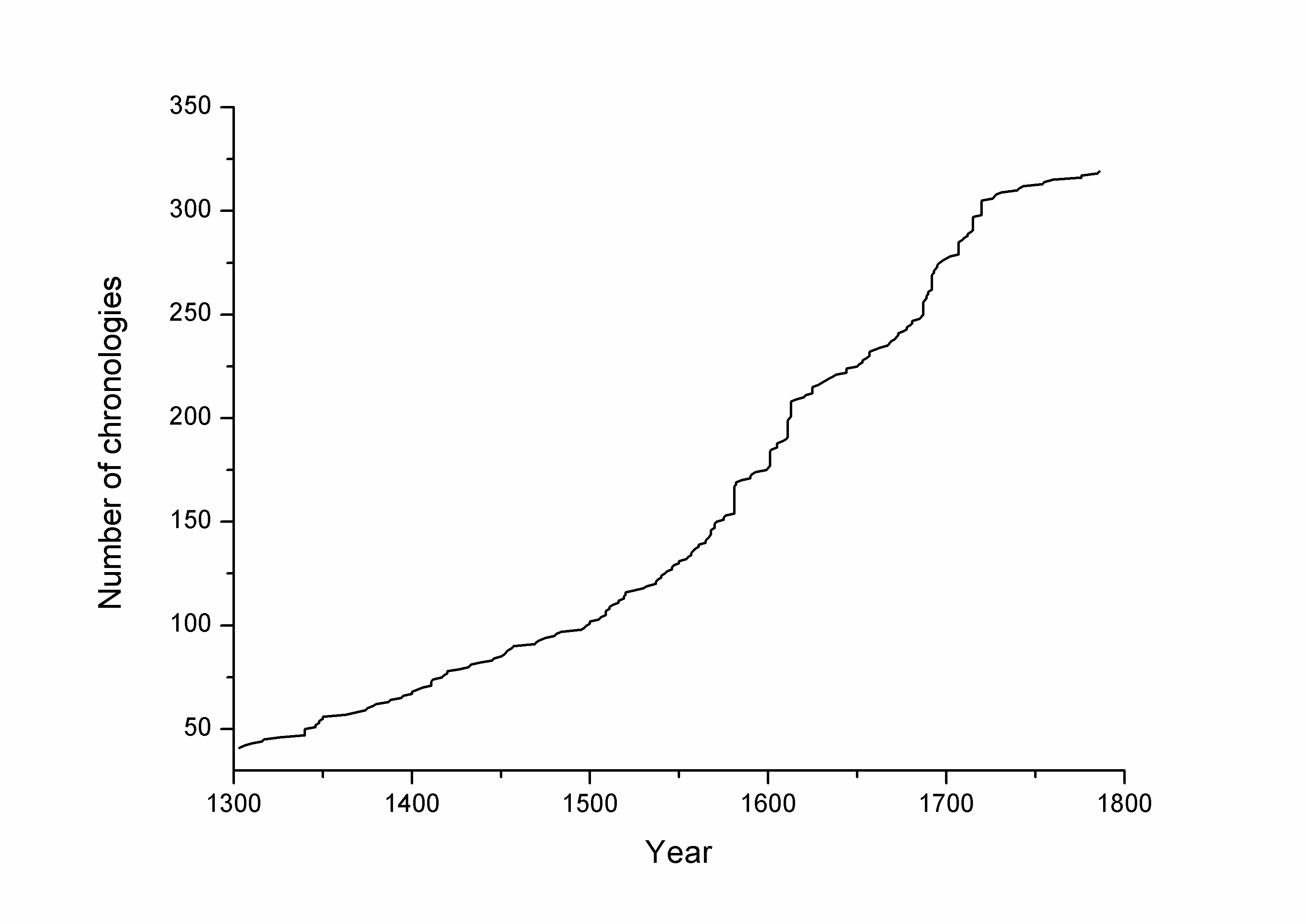

Supplement: Figure S1 — The time-varying number of tree-ring chronologies used in MADA. (TIF) [file pone.0102751.s001.tif]

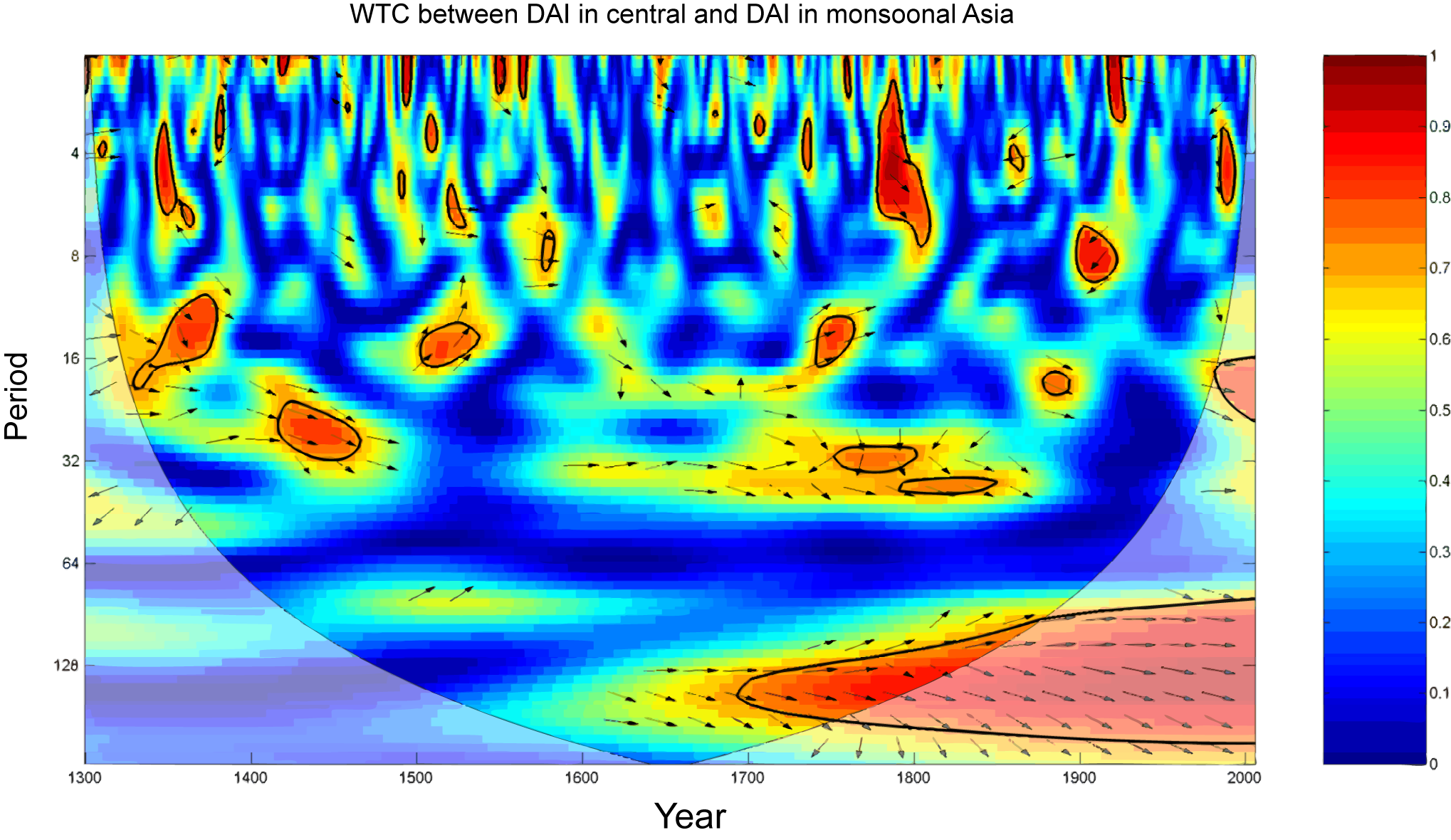

Supplement: Figure S3 — Squared wavelet coherence (WTC) of the drought area index (DAI) between central and monsoonal Asia. The arrows pointing left (right) indicate anti-phase (in-phase) relationship. The areas with significance (p<0.05) relationships are indicated by thick contours. (TIF) [file pone.0102751.s003.tif]
